# Directed Forgetting in Post-Traumatic-Stress-Disorder: A Study of Refugee Immigrants in Germany

**DOI:** 10.3389/fnbeh.2013.00094

**Published:** 2013-08-07

**Authors:** Michaela Baumann, Bastian Zwissler, Inga Schalinski, Martina Ruf-Leuschner, Maggie Schauer, Johanna Kissler

**Affiliations:** ^1^Department of Psychology, University of Konstanz, Konstanz, Germany; ^2^Department of Psychiatry, University of Tuebingen, Tuebingen, Germany; ^3^Center of Excellence for Psychotraumatology, University of Konstanz, Konstanz, Germany; ^4^Department of Psychology, University of Bielefeld, Bielefeld, Germany

**Keywords:** directed forgetting, post-traumatic stress disorder, emotion, dissociation

## Abstract

People with post-traumatic stress disorder (PTSD) often suffer from memory disturbances. In particular, previous studies suggest that PTSD patients perform atypically on tests of directed forgetting, which may be mediated by an altered emotional appraisal of the presented material. Also, a special role of dissociative symptoms in traumatized individuals’ memory performance has been suggested. Here, we investigate these issues in traumatized immigrants in Germany. In an item-method directed forgetting task, pictures were presented individually, each followed by an instruction to either remember or forget it. Later, recognition memory was tested for all pictures, regardless of initial instruction. Overall, the PTSD group’s discrimination accuracy was lower than the control group’s, as PTSD participants produced fewer hits and more false alarms, but the groups did not differ in directed forgetting itself. Moreover, the more negatively participants evaluated the stimuli, the less they were able to discriminate old from new items. Participants with higher dissociation scores were particularly poor at recognizing to-be-forgotten items. Results confirm PTSD patients’ general discrimination deficits, but provide no evidence for a distinct directed forgetting pattern in PTSD. Furthermore, data indicate that, in general, more negatively perceived items are discriminated with less accuracy than more positively appraised ones. Results are discussed in the larger context of emotion and stress-related modulations of episodic memory, with particular focus on the role of dissociative symptoms.

## Introduction

Post-traumatic stress disorder (PTSD) is a possible aftereffect of extremely traumatic life experiences, that is, events which threaten one’s own or others’ lives or physical integrity and to which the individual reacts with intense feelings of fear or helplessness (APA, 1994). The disorder is characterized by symptoms of re-experiencing, avoidance/emotional numbing, and heightened arousal, persisting for a month at minimum.

Memory disturbances are a core feature of PTSD (for a review, see McNally, 2006). They can alternate between recurrent, intrusive recollections, and an inability to recall autobiographical information, including the traumatic experience, a paradox reflected in the diagnostic criteria (APA, 1994). On laboratory tasks, memory deficits in PTSD become apparent as a reduced ability to recall recently studied items in explicit tests and as an increased tendency to produce false memories in free recall (see Brewin et al., 2007) and old–new recognition tests (Bremner et al., 2000; Zoellner et al., 2000; Goodman et al., 2011).

The directed forgetting paradigm (DF; for an overview, see Johnson, 1994) is well-suited to explore cognitive abnormalities in PTSD. It can be used to test for the capability to selectively remember relevant and to forget irrelevant information and to distinguish presented items from similar, but never presented material. In DF tasks, participants are shown items and instructed to remember some of them but forget others. Later, memory is tested for both remember (R) and forget (F) items. Usually, participants are able to reproduce more R- than F-items – the DF effect. Conversely, false memories are typically higher in the F than in the R condition (e.g., Kimball and Bjork, 2002; Zwissler et al., 2011, 2012). In item-method DF, where each single item is followed by an instruction to either remember or forget it, the DF effect is generally attributed to selective encoding, since participants can stop encoding items immediately after receiving F instructions and can selectively rehearse after R instructions. In item-method DF, effects are seen on both free recall and recognition tasks.

Several studies have addressed modulations of item-method DF in clinical populations (for a review, see Geraerts and McNally, 2008). In particular, the hypothesis has been examined that traumatized individuals [particularly survivors of childhood sexual abuse (CSA)] use an avoidant or dissociative encoding style and are therefore better able to disengage from threatening events resulting in particularly poor encoding of emotionally negative F-items and larger DF effects (Terr, 1994). However, subsequent studies with traumatized participants in general or PTSD patients were inconsistent (see Geraerts and McNally, 2008).

For instance, Cloitre et al. (1996) found a larger recall advantage for R- versus F-items in individuals with borderline personality disorder and parental abuse histories, relative to borderline personality disorder without abuse histories or healthy controls. However, in a subsequent study, participants with borderline personality disorder, unlike controls, exhibited superior recall for negative F words, providing no evidence for participants’ avoidant encoding of threatening information (Korfine and Hooley, 2000).

McNally and his colleagues investigated in several DF studies whether individuals with repressed, recovered, or continuous memories of CSA performed differently from participants without a history of CSA (e.g., McNally et al., 1998, 2001, 2004). Under the avoidant encoding hypothesis, CSA survivors, particularly those who claimed to have forgotten their abuse (i.e., participants with repressed or recovered memories), should forget more trauma-related words than participants with continuous memories or those without a history of CSA. Yet overall, participants reporting repressed or recovered memories of CSA did not differ from the other groups in DF performance, providing no evidence for avoidant encoding.

Zoellner et al. (2003) investigated DF in PTSD patients and healthy controls under the assumption that the current mood state affects DF patterns. They induced either a dissociative or a serene mood in PTSD patients and controls before the DF task. Although a standard DF effect occurred in the serenity condition, contrary to predictions this effect was completely absent in the dissociation condition, suggesting that, if anything, the dissociative state reduced rather than increased directed forgetting. Moreover, the PTSD and control groups recalled comparable numbers of threat-related items (a finding similar to McNally et al., 1998). Thus, the study neither provided evidence for avoidant nor for intrusive encoding of threatening information in PTSD. DePrince and Freyd (2001, 2004) argued that dissociation may facilitate DF performance under divided attention, in particular. Indeed, in a pre-selected student population, high-dissociative students recalled fewer trauma-related and more neutral items under divided attention conditions than did low-dissociative students. However, replications by other authors failed (McNally et al., 2005; Devilly et al., 2007; but see DePrince et al., 2007; Devilly and Ciorciari, 2007 for critical discussions).

Some evidence that traumatized individuals may indeed be better at forgetting particularly threatening material and that dissociative symptoms may play a special role comes from studies on participants with acute stress disorder (ASD; Moulds and Bryant, 2002, 2005): ASD patients recalled fewer trauma-related F words compared to controls and trauma-exposed participants without ASD. The authors suspected a modulatory role of dissociative symptoms, which are essential for the diagnosis of ASD but not PTSD. According to Moulds and Bryant, these might have caused superior forgetting in the ASD group.

So far, the evidence on DF patterns in traumatized individuals is very heterogeneous. Regarding the impact of emotional stimuli, one theory posits that traumatized individuals are superior at forgetting in general and at forgetting emotional or threatening information in particular. However, some of the above cited studies point to reduced DF, especially for emotional material. Similarly, Zwissler et al. (2012) found, in an item-method DF study on Ugandan civil war victims with and without PTSD, a DF effect in the control but not in the PTSD group. Furthermore, PTSD participants produced more false alarms than controls and rated the stimuli as significantly more arousing than Non-PTSD participants. Higher arousal ratings were associated with a reduced DF effect, suggesting that the attenuation of DF in PTSD was mediated by increased subjective arousal, in line with the finding that healthy participants in general have reduced DF for emotional information on recognition tasks (Hauswald et al., 2011; Zwissler et al., 2011).

Still, a role for a dissociative processing style resulting in more DF has been identified by some studies (Terr, 1994; DePrince and Freyd, 2001, 2004; Moulds and Bryant, 2002, 2005). Moreover, recently hyper-aroused and dissociative PTSD sub-types have been suggested. These sub-types are supposed to differ in their physiologic and psychological response patterns to trauma reminders, the dissociative sub-type being characterized by emotional numbing, in particular (Schauer and Elbert, 2010). If so, different ad-mixtures of these sub-types in different memory experiments may have contributed to the inconsistent results in PTSD.

Overall, emotional content of the stimuli (e.g., valence and arousal), the trauma relevance of the stimuli, as well as the patients’ dissociative symptoms have been suggested to affect DF in PTSD, but so far no study has considered these factors simultaneously. The present study investigates the DF performance of asylum seekers with and without PTSD in Germany, specifically examining dissociative symptoms and subjective stimulus appraisal as possible mediating factors. Because of their frequent exposure to traumatic events during war and political persecution, PTSD prevalence rates of up to 40% have been reported for asylum seekers (Gäbel et al., 2006; see also Lindert et al., 2008). However, the cognitive consequences of this fact are largely unexplored. We addressed DF patterns in this population in a language-free pictorial task, followed by arousal and valence ratings of the stimuli. Group differences in overall recognition memory (hits, false alarms, discrimination accuracy, response bias) and directed forgetting were investigated and the relationship between DF, participants’ dissociation scores and stimulus appraisals were examined.

## Materials and Methods

### Participants

Twenty-five migrants and refugees took part in the study, 12 being diagnosed with PTSD and 13 serving as controls.

For the PTSD group, individuals currently investigated or treated at the University of Konstanz Outpatient Clinic for Refugees (*Psychologische Forschungs- und Modellambulanz für Flüchtlinge*) were invited to participate. They all met DSM-IV criteria for PTSD (APA, 1994), as previously assessed with the Clinician-Administered PTSD Scale (CAPS; Blake et al., 1995) in the course of standard diagnostic procedures at the outpatient department. The PTSD group consisted of eight women and four men. Five participants were from African countries, three from Turkey, two from Kosovo, and one each from Iran and Iraq. On average, they were 32.3 years old (SD = 9.28) and had received 7.67 years of education (SD = 5.50).

The control group was partly recruited at the outpatient clinic (controls or interpreters of previous studies; *n* = 6) and partly through information and language courses for migrants (*n* = 7). All were screened for PTSD using the Post-traumatic Diagnostic Scale (PDS; Foa et al., 1997), and none of them met DSM-IV criteria (APA, 1994). Efforts were made to match the control group to the PTSD group: the controls had migrated from countries similar to the PTSD group’s (five were from African countries, two from Iran, and one from Iraq, Turkey, Russia, Belarus, Romania, and Hungary, respectively) and neither differed significantly from the PTSD group in age [*M* = 34.5, SD = 11.2; *t*(23) = 0.53, *p* = 0.60^1^] nor in gender distribution [10 women versus three men; *L*χ^2^ (1) = 0.33, *p* = 0.67]. However, they had received formal education approximately 7 years longer than had the PTSD group [*M* = 14.9, SD = 3.19; *t*(17) = 3.97, *p* = 0.001].

### Stimuli, design, and procedures

After giving informed consent, participants were administered the Dissociative Experiences Scale (DES; Bernstein and Putnam, 1986). The DES is a self-assessment questionnaire measuring dissociative phenomena like amnesia, absorption, and alienation. Its 28 items describe experiences such as, “Some people have the experience of finding new things among their belongings that they do not remember buying” and, “Some people have the experience of feeling that other people, objects, and the world around them are not real.” For each experience, participants rate the frequency of occurrence on a scale from 0 to 100, and the mean of these item scores constitutes the DES score. The DES possesses good reliability and validity (e.g., Frischholz et al., 1990; Dubester and Braun, 1995). For participants who neither spoke German nor English fluently, interpreters translated during the whole session.

**Figure 1:**
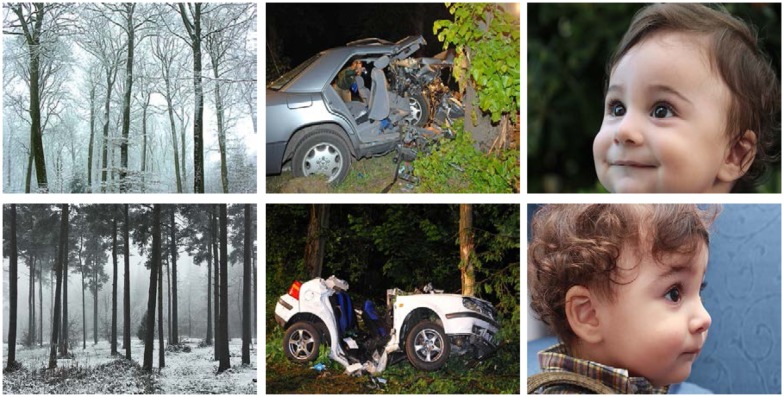
**Three exemplary pairs of used target and distracter pictures illustrating the range of emotional intensity and valence covered by the stimuli as well as target – distracter similarity**.

Completion of the DES was followed by the DF task. Here, two sets of 28 complex photographs served as stimuli. The pictures showed scenes of daily life, such as landscapes, vehicles, or people (see Figure 1 for examples). The sets were paired such that every picture in one set had a similar counterpart in the other set. In the learning phase, the 28 pictures of one set were presented on a computer screen in random order for 4000 ms each. Every picture was followed by a 2000 ms instruction indicating whether participants should memorize the picture (instruction “MMM” for the German word “merken”) or forget it (“VVV” for “vergessen”). Then, a fixation cross appeared for 2000 ms before the next photograph was presented. After the learning phase, participants worked on “Tangram” puzzles for 5 min as a distracter task. In the ensuing recognition phase, the 28 pictures of the learning set were presented randomly intermixed with the 28 photographs of the second set, which served as lures. Each picture was shown for 1000 ms and participants made old–new decisions by pressing the right (for *old*) or left (for *new*) mouse button within a time limit of 4000 ms. Participants were informed that new pictures could look quite similar to old ones, and, importantly, that their old–new decisions should be independent of the original instructions associated with the pictures (i.e., participants were supposed to chose *old* if they recognized pictures regardless of whether they had been designated as R or F).

Afterward, participants rated each of the 56 photographs on the dimensions valence, arousal, and trauma relevance: they indicated on nine-point scales whether they found a picture pleasant or unpleasant (valence), arousing or quiet (arousal), and whether it reminded them of their own traumatic experiences (trauma relevance). Controls were additionally screened for PTSD with the PDS at the end of the session before they – like PTSD participants – received a compensation of 20 €.

### Statistical analysis

Statistical calculations were carried out with SPSS Version 17.0. In a first step, recognition data were statistically analyzed by means of mixed-model ANOVAs with response type and instruction as within factors (hits R, hits F, false alarms R, false alarms F) and group as between factor (PTSD versus control). Effects are reported as significant at *p* < 0.05. In a second step, group differences in picture appraisals and dissociative symptoms were assessed and their correlative relationship explored. Finally, the influence of picture appraisals and dissociative symptoms on memory performance was addressed by entering mean valence, arousal, and trauma relevance ratings as well as DES scores individually into the ANOVA model as covariates. Furthermore, targeted analyses investigated the linear relationships between recognition accuracy for F and R items and valence, arousal, trauma relevance as well as dissociative symptoms.

## Results

### Recognition memory and directed forgetting

Table 1 presents mean hit and false alarm rates in the R and F conditions for the sample as a whole as well as for the two subgroups. Participants were included in the analyses if the number of pictures they designated as old was within one standard deviation around the mean of pictures designated as old across participants (*M* = 28.9, SD = 7.31; resulting in *n* = 20^2^). For hits, a significant main effect of group [*F*(1, 18) = 6.14, *p* = 0.023] indicated that the control group produced more hits than the PTSD group. Regarding false alarms, there was a significant main effect of instruction [*F*(1, 18) = 7.05, *p* = 0.016], that is, participants produced more false alarms for F than R items and an effect of group [*F*(1, 18) = 4.23, *p* = 0.054], indicating that PTSD participants produced more false alarms than controls.

**Table 1:** **Mean hit and false alarm rates in the R and F conditions for the whole sample as well as separately for the PTSD and control group**.

	Hits R (SD)	Hits F (SD)	False alarms R (SD)	False alarms F (SD)
Overall (*n* = 20)	0.77 (0.17)	0.77 (0.18)	0.23 (0.17)	0.30 (0.17)
PTSD (*n* = 8)	0.70 (0.19)	0.65 (0.20)	0.30 (0.20)	0.39 (0.14)
Control (*n* = 12)	0.82 (0.15)	0.85 (0.12)	0.18 (0.13)	0.24 (0.17)

**Figure 2:**
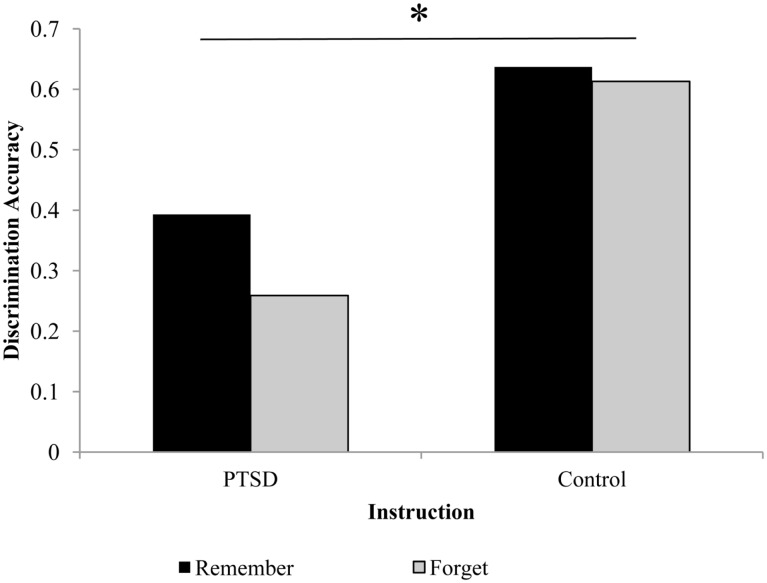
**Discrimination accuracy for the remember (R) and forget (F) conditions in the PTSD and control group illustrating both the group difference in recognition accuracy and the directed forgetting pattern**.

Next, hits and false alarms were considered contemporaneously, differentiating between discrimination accuracy (Pr = hits – false alarms) and response bias [Br = false alarms/(1–Pr)]. In terms of discrimination accuracy, a trend-level effect for instruction was observed [*F*(1, 18) = 3.99, *p* = 0.061; see Figure 2]: participants tended to show better discrimination accuracy for R than for F-items, that is, a DF effect. Moreover, the control group’s discrimination accuracy was higher than PTSD group’s, as demonstrated by a main effect of group [*F*(1, 18) = 6.27, *p* = 0.022]. There was no significant interaction of instruction and group [*F*(1, 18) = 1.95, *p* = 0.18]. Although PTSD patients and controls did not differ significantly in DF performance, visual inspection suggests that the DF effect was mainly carried by the PTSD group. For recognition bias, no significant main effects or interactions were found and the two groups did not differ significantly in that measure [*F*(1, 18) = 0.46, *p* = 0.51].

### Picture appraisal and dissociative symptoms

Overall, participants rated the pictures on the respective nine-point scales as somewhat negative (*M* = 3.70, SD = 0.81), of average arousal (*M* = 5.30, SD = 1.29), and not very trauma relevant (*M* = 3.56, SD = 1.79). Comparing the two groups, there was a trend for the PTSD group to rate the pictures as more negative than the control group [dimension of valence; *t*(18) = 1.94, *p* = 0.068]. The ratings regarding the other dimensions were similar for the two groups, neither differing in arousal [*t*(18) = 1.24, *p* = 0.23] nor in trauma relevance [*t*(18) = −0.61, *p* = 0.55].

**Figure 3:**
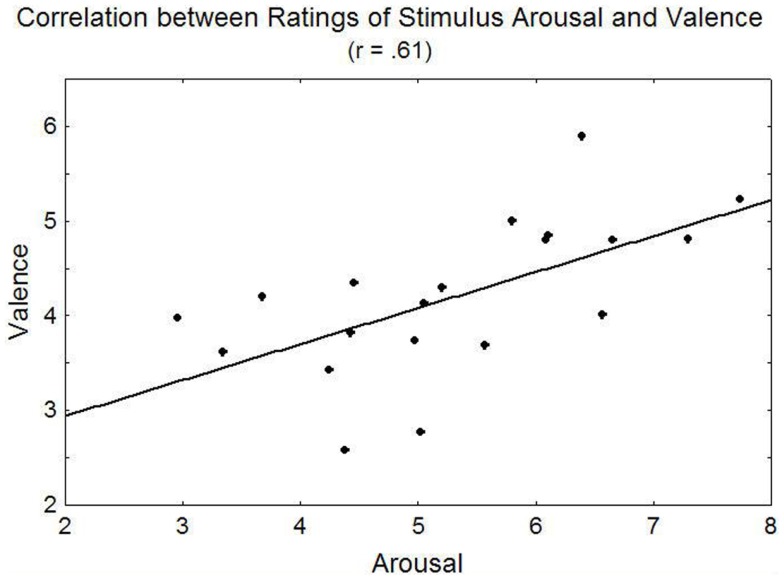
**Relationship between valence and arousal ratings in the present sample**.

Considering dissociation, the PTSD group had a mean DES score of 32.6 (SD = 7.32), which was much higher than the control group’s [*M* = 11.3, SD = 11.7, *t*(18) = 4.55, *p* < 0.001]. For two participants, the DES could not be administered. These participants’ DES scores were therefore substituted by their scores on a similar dissociation questionnaire^3^. Overall, dissociation scores were negatively correlated with ratings of picture arousal (*r* = −0.44, *p* = 0.05) and in tendency also valence (*r* = −40, *p* = 0.08) and the relationship between valence and arousal ratings was well described by a linear relationship [*r* = −0.61, *p* < 0.01; *r*^2^ = 0.37, *F*(1, 18) = 10.56, *p* < 0.01] indicating that on average the more negative pictures were not rated as arousing but more positive pictures were rated as also more arousing. The linear relationship between valence and arousal observed in this sample is illustrated in Figure 3.

### Recognition memory, picture appraisal, and dissociative symptoms

**Figure 4:**
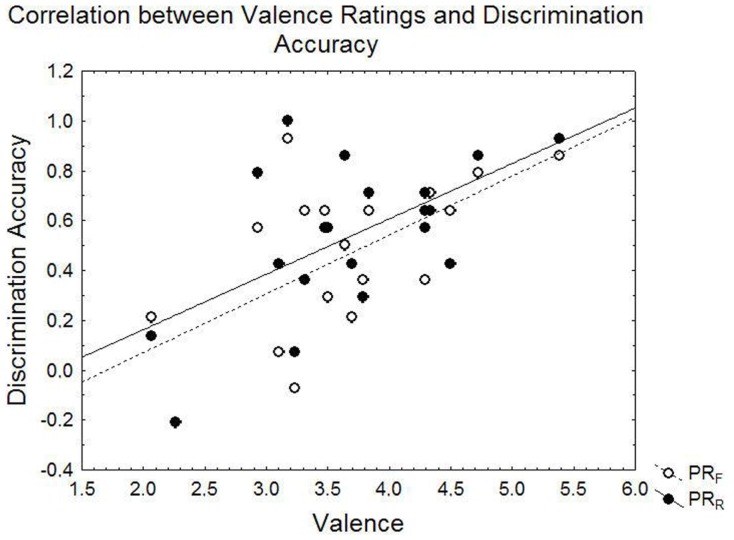
**Illustration of the linear correlation between mean valence ratings and discrimination accuracy (Pr)**. For valence ratings, higher numbers indicate more positive ratings.

ANCOVA revealed that discrimination accuracy differed depending on the perceived valence of the pictures [*F*(1, 17) = 6.65, *p* = 0.020]. Pearson correlations indicated a significant positive association between mean picture valence and discrimination accuracy (*r* = 0.59, *p* = 0.007 for R items; *r* = 0.61, *p* = 0.004 for F-items). As demonstrated in Figure 4, the more positive an individual rated the pictures, the higher the discrimination accuracy score. The two groups contributed similarly to this effect: in the PTSD group, the relationship between Pr and valence was *r* = 0.56, *p* = 0.15 for F-items and *r* = 0.63, *p* = 0.09 for R-items. In the control group it was *r* = 0.53, *p* = 0.08 for F-items and *r* = 0.46, *p* = 0.13 for R-items.

**Figure 5:**
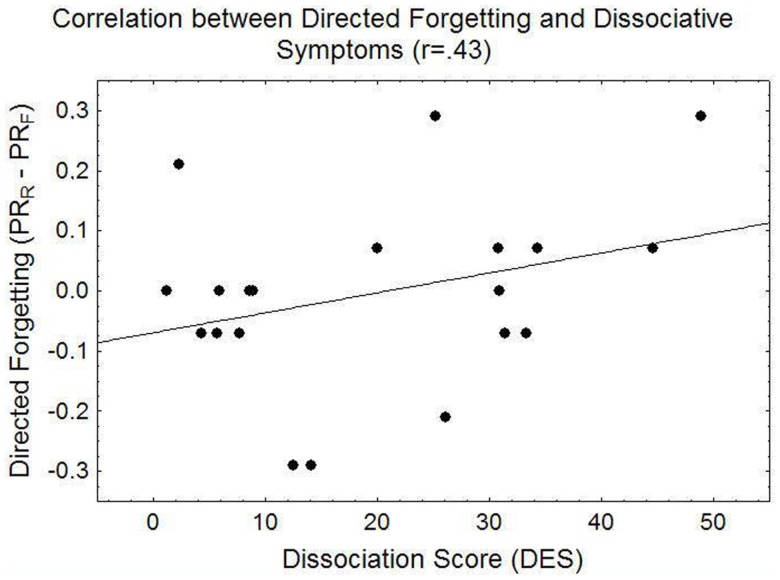
**Illustration of the linear correlation between the magnitude of DF (in terms of recognition accuracy Pr) and scores on the dissociative experience scale (DES)**.

**Figure 6:**
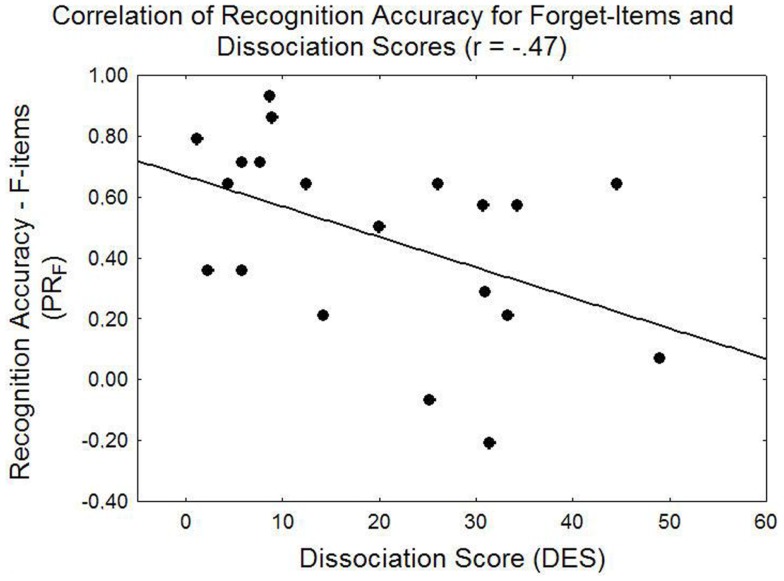
**Illustration of the linear correlation between recognition accuracy for to-be-forgotten items and dissociation scores**.

Adding mean arousal or trauma relevance ratings, or DES scores as covariates yielded no significant main effects. However, targeted correlation analyses revealed a trend for a positive relationship between DF magnitude (Pr_R_ – Pr_F_) and DES scores (*r* = 0.43, *p* = 0.061), the numerical value of which was higher in the PTSD (*r* = 0.60) than control group (*r* = 0.18, both n.s.). In particular, DES scores were negatively correlated with Pr_F_, the classification accuracy for F-items (*r* = −0.47, *p* < 0.05), indicating reduced recognition accuracy for F-items, but not R-items (*r* = −0.23, *p* = 0.33) in people with higher DES scores. Figure 5 displays the overall relationship between directed forgetting and dissociation scores and Figure 6 specifically illustrates the relationship between recognition accuracy for F-items and dissociation scores.

## Discussion

This study investigated item-method DF in a culturally heterogeneous group of traumatized refugees with PTSD, focusing on the role of possible mediating factors such as dissociative symptoms and stimulus appraisal. To this end, an item-method DF task with complex pictorial stimuli was used. Participants’ dissociative symptoms and subjective appraisals of stimulus valence, arousal, and trauma relevance were assessed, and these variables’ relationship to DF patterns was investigated.

Overall, controls produced more hits than PTSD patients, whereas PTSD patients produced more false alarms than controls, resulting in worse discrimination accuracy in the PTSD group. Consistent with typical DF patterns in recognition memory designs, across groups more false alarms were generated for F-lures than for R lures. Also, the more positively a picture was evaluated, the better it was discriminated. Moreover, participants with more dissociative symptoms had worse recognition of to-be-forgotten pictures and rated the pictures as less arousing and in tendency also more negative.

In the following, we will discuss these results in relation to extant literature and consider further suggestions emerging from this study: the higher false alarm rate in the PTSD group is consistent with previous reports of higher susceptibility to false memories in traumatized individuals (Bremner et al., 2000; Zoellner et al., 2000; Goodman et al., 2011). Considering hits and false alarms together, the control group’s discrimination accuracy was higher than the PTSD group’s, reflecting PTSD patients’ memory impairment (e.g., Jelinek et al., 2006; McNally, 2006; Brewin et al., 2007) and extending this finding to a ethnically diverse population of immigrant refugees.

The directed forgetting instruction affected memory performance in that a significantly higher number of false alarms for F than R items (Kimball and Bjork, 2002; Zwissler et al., 2011, 2012) and a trend toward higher discrimination accuracy for R- than F-items were found. However, the two groups did not differ significantly in DF performance and if anything, the DF effect appeared to be carried more by the PTSD than by the control group. Several factors might explain this pattern: first, statistical power may have been insufficient as in between-group designs, 26 participants are considered necessary even for large effect sizes, given an α-level of 0.05, to achieve a statistical power of 0.8 (Cohen, 1992). For medium and small effect sizes, 64 and 393 participants would be needed, respectively, clearly many more than took part in the present study and produced useable data. Second, to avoid floor effects in the patient group, this study contained relatively few items and item presentation (4000 ms) was longer than in our previous experiments (Hauswald and Kissler, 2008; Hauswald et al., 2011; Zwissler et al., 2012). This may have fostered encoding of both R- *and* F-items in the non-memory impaired control group, thereby reducing DF. Studies that found a reduction of DF effects with increasing item-cue intervals, support this explanation (Wetzel and Hunt, 1977; Hourihan and Taylor, 2006).

The near ceiling results in the control group could also be attributable to the use of pictorial material. Although several studies (Hauswald and Kissler, 2008; Hauswald et al., 2011; Nowicka et al., 2011; Zwissler et al., 2011, 2012) have found robust DF effects using complex pictures, effects tend to be smaller than those typically found for words (Quinlan et al., 2010). However, pictorial stimuli allow for investigation of culturally and linguistically heterogeneous groups. In the present sample there was no single language all participants spoke.

Regarding the influence of stimulus appraisal on memory, valence ratings were significantly correlated with discrimination accuracy: the more positively the picture set was evaluated, the more accurately the pictures were discriminated. This result fits well with a study on intentional forgetting in children with PTSD that found significantly higher recognition accuracy for positive than negative words (Yang et al., 2010). In the present study PTSD patients tended to rate the pictures more negatively than did controls, which might contribute to their impaired discrimination accuracy. In general, memory for emotionally negative pictures is based more on gist than on detail (Adolphs et al., 2001), but the present design required encoding of details for successful discrimination.

The present study does not indicate that PTSD patients as such have larger DF effects, which is in line with other researchers’ failure to observe differences between traumatized participants and controls (e.g., McNally et al., 1998, 2001, 2004). However, participants with higher dissociation scores had lower discrimination accuracies specifically for to-be-forgotten items. Indeed, under the avoidant encoding hypothesis individuals with high dissociation scores should be superior forgetters and thus show reduced memory for F-items. This pattern could be taken as tentative support for a special role of dissociative symptoms in directed forgetting. However, given the small sample and the controversy in the literature (cf. Cloitre et al., 1996; DePrince and Freyd, 2001, 2004; McNally et al., 2001, 2004), this finding needs extension and replication. Participants with higher dissociation scores also rated the stimuli as less arousing and somewhat more negative. This is in line with emotional numbing in dissociation (Frewen and Lanius, 2006) and could also account for the unusual linear relationship between valence and arousal found in the present data. Whereas typically both more negative and more positive stimuli are rated as more arousing, here, this held only for positive stimuli.

PTSD patients have been suggested to differ according to their physiologic and psychological response patterns to trauma reminders, resulting in hyper-aroused and dissociative sub-types (Schauer and Elbert, 2010) and the present results point to possible mnemonic consequences. Also, Moulds and Bryant (2002, 2005) founder larger DF effects in ASD, whose diagnosis requires high-dissociative symptom scores. Although challenging to conduct, a comparison of two large groups of PTSD patients who differ solely in their dissociative versus hyperarousal symptoms would provide a crucial test.

The previous finding of reduced DF for more arousing stimuli, concomitant with higher arousal ratings in PTSD, was not replicated in this study (Zwissler et al., 2012). Whereas several studies found a reduction of item-method DF for arousing stimuli in healthy participants (Yang et al., 2010; Hauswald et al., 2011; Nowicka et al., 2011; Zwissler et al., 2011, 2012), so far no study has systematically assessed the influence of valence and arousal on item-method DF across a wide range of arousal levels in large groups of PTSD patients. As suggested by the present data, emotional appraisal may vary with clinically relevant symptoms such as dissociation. Moreover, potential cultural variations in emotional appraisal are virtually unexplored. Future studies, using a broader range of stimuli, can shed more light on the relationship between valence, arousal, and DF in PTSD. In order to avoid clinical complications such as flashbacks or dissociative fainting during the experiment, the present experiment only used stimuli that were of relatively moderate valence and arousal, limiting statistical power to detect an effect on memory performance. This restriction probably also reduced our ability to identify any influence of “trauma relevance” on our data. Although participants perceived some variation of emotional content in the stimuli, they did not find them particularly representative of their own, often quite atrocious, experiences.

Taken together, results demonstrate reduced discrimination accuracy in PTSD patients, resulting from fewer hits and more false alarms. Data also reveal worse discrimination accuracy for more negatively evaluated stimuli. Participants with more dissociative symptoms exhibited altered stimulus appraisal, rating the stimuli as less emotionally intense. Importantly, individuals with high dissociation scores may indeed be better at directed forgetting. However, this last finding needs further scrutiny in a larger study, although large samples are difficult to obtain in this population. Traumatized patients typically have many problems related to their trauma, traumatized immigrants in particular also have many problems related to social and cultural factors, which, as such reduce their motivation to take part in scientific studies as well as reduce data quality and interpretability.

Despite these limitations, research on underprivileged samples that receive little attention from scientists and health-care providers, such as refugees with PTSD, is important for science and practice: systematic investigation will reveal typical cognitive phenomena and facilitate their scientific explanation. Identification of functional problems can aid the development of effective treatments. In refugees and migrants from war-torn societies, extremely traumatic experiences and resulting psychological impairments are quite frequent (Gäbel et al., 2006; Kinzie, 2006). Their investigation is essential to understand the characteristics, impairments, and needs of refugees and migrants in order to eventually provide help. The current study took one step toward this goal.

## Conflict of Interest Statement

The authors declare that the research was conducted in the absence of any commercial or financial relationships that could be construed as a potential conflict of interest.

## Footnotes

^1^Before comparing means, equality of variances was examined using Levene’s test. For unequal variances, corrected *t*-values and degrees of freedom are presented.

^2^For the five excluded participants (four PTSD patients and one control), it has to be assumed that instructions were not understood. For example, one participant designated 53 of the 56 pictures in the recognition test as old (when in fact 28 were old); including these extreme “overestimators” would have inflated false alarm rates. Another participant, on the contrary, designated only 13 pictures as old; here, the inclusion of these “underestimators” would have reduced hit rates artificially, without shedding more light on the difference between R- and F-items, which was the main research question in this study.

^3^This questionnaire called *Current Dissociation* was developed in the outpatient center and, similarly to the DES, asks for the occurrence of typical dissociative symptoms like blurred vision, out-of-body experiences, etc., in daily life. Since answers are rated on four-point scales, the mentioned two patients’ test scores were transformed into a percentage rate (points reached/points possible) to ensure comparability with DES scores.
